# Evaluation of Copper-Induced Cytotoxicity and Transcriptomic Change Using a RTgill-W1 Cell Line as an Alternative Replacing Fish Test

**DOI:** 10.3390/toxics13110924

**Published:** 2025-10-28

**Authors:** Jin Wuk Lee, Ilseob Shim, Kyunghwa Park

**Affiliations:** Hazard Management Division, National Institute of Chemical Safety, Incheon 404-708, Republic of Korea; similseob@korea.kr (I.S.); mirrorpark@korea.kr (K.P.)

**Keywords:** RTgill-W1 cell line, biomarker, cytotoxicity, RNA-sequencing, RT-PCR, copper

## Abstract

The RTgill-W1 cell line serves as an alternative for acute fish toxicity testing. This study aims to study the reliability of the RTgill-W1 cell line in copper cytotoxicity using transcriptomic analysis followed by comparison with existing literature. As a result, the study found that the average EC50 (375 μg/L ± 181 μg/L) in cell viability was similar to previous literature results (0.093–530 μg/L), suggesting the system’s reliability as an alternative. The transcriptome changes of the RTgill-W1 cell line caused by copper exposure are supported by the existing literature on individual fish. For example, osmoregulatory disturbances, regulation of Na^+^/K^+^-ATPase activity, oxidative stress, apoptosis, energy metabolism alterations, metal detoxification, and chaperone protein expression were found in the RTgill-W1 cell line in response to copper exposure, indicating the utility of this cell line for transcriptome analysis. Finally, through RT-PCR confirmation and literature analysis, this study suggests that *sirtuin 1*, *sirtuin 4*, *Na^+^/K^+^-ATPase*, *aifm4*, *bcl2*, *carbonic anhydrase*, *hsp70*, *hsp30*, and other biomarkers could be used for detecting copper stress in aquatic organisms. This study is helpful for understanding the toxicity mechanism of copper and can be referred to as scientific data for regulating copper release into the aquatic environment.

## 1. Introduction

As concern for animal protection increases, the demand for developing alternative toxicity tests that replace animal testing continues to grow. One well-known alternative is cellular toxicity testing [1]. Recently, in the field of aquatic toxicity testing, the RTgill-W1 cell line derived from the gills of rainbow trout (*Oncorhynchus mykiss*) has attracted attention. In *OECD Test Guideline (TG) 249*, it is used as a test system to replace fish acute toxicity tests for chemical effects [1]. Additionally, several studies have studied the reliability of this test system in predicting chemical toxicity comparable to those obtained from in vivo experiments with fish [1,2]. Therefore, applying the RTgill-W1 system to copper toxicity assessment and comparing its results with existing in vivo data are essential for understanding its applicability as an alternative test system.

Molecular-level responses of organisms can serve as early warning signals for adverse chemical effects and are crucial for understanding the mechanisms underlying these effects. To analyze molecular responses effectively, transcriptome-level gene expression analysis has been widely used in chemical effect studies. Techniques such as RNA sequencing (RNA-seq) and RT-PCR are commonly employed [3,4,5]. RNA-seq monitors transcriptome changes in response to chemical exposure and is a powerful method for comprehensively identifying affected molecular pathways [6,7]. However, few studies have utilized this technique for investigating metal toxicology in fish [8]. RT-PCR, which analyzes specific RNA quantities using a specific primer, offers higher specificity and sensitivity compared to RNA-seq. Thus, this study employs both RT-PCR and RNA sequencing to evaluate biomarker responses at the gene expression level. Transcriptomic information analyzed using these tools can be a key data for establishing adverse outcome pathways (AOP) that play an important role in an alternative toxicity test as a conceptual framework to predict individual or higher level chemical toxicity based on molecular level effect information [9]. In this context, application of RT-PCR and RNA sequencing to toxicity tests with the RTgill-W1 cell line would be an effective alternative approach to chemical safety assessment.

Copper (Cu) is an essential element in biological systems, serving as a vital nutrient involved in various metabolic processes at low concentrations. It is widely used in anthropogenic products, such as disinfectants, water purifiers, algae- and fungicides, nematocides, molluscicides, and anti-bacterial and anti-fouling agents [10]. However, excessive exposure to copper beyond biological needs can have detrimental effects, including lethality, oxidative stress, apoptosis, immune system dysfunction, osmoregulatory failure, energy imbalance, reproductive toxicity, and metabolic disturbances in fish and other species [11,12,13,14,15]. Consequently, controlling copper concentrations is a major concern for many national and international regulatory agencies.

Copper accumulates in various fish organs, including the gills, kidney, brain, liver, and skeletal muscle [16]. Among these, the gills are the primary target of copper exposure and play a critical role in first-pass metabolism for xenobiotics [4]. This suggests that gill cell lines are suitable test systems for toxicity assessments. Additionally, *O. mykiss* is known for its sensitivity to environmental stressors [17]. However, few studies have investigated multi-biological-level copper effects using RWgill-W1 cell lines. Therefore, in this study, a cell line derived from the gills of *O. mykiss* (RWgill-W1) was chosen for copper toxicity testing.

This study aims to evaluate the adverse effect of copper using the RT-gill-W1 cell line and test its reproducibility compared to existing literature as an alternative test system to the fish toxicity test. Specifically, it seeks to assess the predictive accuracy of the RTgill-W1 cell-based toxicity test compared to traditional fish acute toxicity tests, and to analyze molecular responses at the transcriptome level.

## 2. Materials and Method

### 2.1. Materials

RTgill-W1 cells (CRL-2523, ATCC, Manassas, VA, USA), QIAzol^®^ Lysis Reagent (Cat. No. 79306), RNeasy^®^ Mini Kit (Qiagen Co., Venlo, The Netherlands), Illumina TruSeq Stranded mRNA Sample Prep Kit #RS-122-2101 (Illumina, Inc., San Diego, CA, USA). TECAN AT/Spark Microplate reader (TECAN Trading AG, Männedorf, Switzerland), Allegra X-30R centrifuge (Beckman coulter Inc., Brea, CA, USA), Biological safety cabinet (CHC Lab Co., Ltd., Daejeon, Republic of Korea). Copper solution (Cat no. ICP-15N-1, AccuStandard^®^, Inc., New Haven, CT, USA). Inductively coupled plasma mass spectrometry (ICP-MS, 7800 ICPMS, Agilent Technologies, Santa Clara, CA, USA).

### 2.2. Fish Cell Line Acute Toxicity Test

The acute toxicity test for copper was conducted according to *OECD Test Guideline No. 249*, with some modifications. The RTgill-W1 cell line was purchased from ATCC (CRL-2523, ATCC, Manassas, VA, USA). Briefly, harvested cell lines cultured in 75T cell culture flasks were seeded into 24-well plates, with 350,000 cells per well. Copper standard solution of 1 mg/L was serially diluted from one stock solution by 2.5 fold with L15 exposure medium, and then the existing L15 cell culture medium was replaced with the copper solution. The L15 exposure medium and culture medium were used according to OECD tg 249. Copper in the L15 exposure medium was prepared at concentrations of 1 mg/L, 400 μg/L, 160 μg/L, 64 μg/L, 25.6 μg/L, and 10.24 μg/L, and applied to RTgill-W1 cells. Samples of the test solution were collected just before the test initiation and after 24 h, and concentrations were analyzed using ICP-MS. For samples treated with copper of 1 mg/L, 160 μg/L, 25.6 μg/L, and 10.24 μg/L, 0.2 g of the test solution was sampled. Since the test chemical solution was made in serial dilution from one stock solution, we thought that just four samples were sufficient for exposure chemical stability analysis. Prior to analysis, samples were treated with hydrogen peroxide (0.5 mL) and nitric acid (10 mL). The mixture was analyzed via ICP-MS under the following conditions: 1550 W RF power, 0.7 L/min nebulizer gas flow rate, 15 L/min plasma gas flow rate, and 0.9 L/min auxiliary gas flow rate. The LOQ, R^2^, IDL, and MDL for copper analysis were 0.4 mg/kg, 0.999, 0.04 μg/L, and 0.1 mg/kg, respectively. After the test, each well was stained with dyes in the following order: Resazurin (Alar), CFDA-AM (CfD), and Neutral Red (Red). Endpoint measurements were conducted using fluorescence at 485 nm (excitation) and 535 nm (emission) for CFDA-AM, at 540 nm (excitation) and 595 nm (emission) for alamarBlue™ and at 540 nm (excitation) and 670 nm (emission) for Neutral Red. All procedures adhered to *OECD Test Guideline No. 249*. The LC_50_ value was calculated using probit analysis with BioStat™ 2009 (AnalystSoft Inc., Vancouver, BC, Canada). Cell line morphology was observed after 24 h exposure was finished and before dye treatment was conducted. At this time, morphology was observed with a microscope at 200 fold magnification (Eclipse Ti2, Nikon, Tokyo, Japan) using software NIS-Elements AR 5.21.00 64-bit (ver 4.0).

### 2.3. RNA Sequencing Analysis

Based on the cell viability test results, 64 (sublethal concentration) and 400 μg/L (near to EC50 value) copper were chosen as a test concentration. Copper standard solution was serially diluted with L15 exposure medium, resulting in 64 and 400 μg/L copper solution. Then existing L15 cell culture medium was replaced with the prepared copper solution. 1 × 10^6^ cells in 25T cell culture flask were treated with the copper solution of 64 or 400 μg/L for 24 h. At every concentration, the samples were replicated three times. Then, cells were harvested and QIAzol reagent added to the cell pellet, being subsequently frozen at −80 °C until RNA analysis.

For total RNA extraction, QIAzol reagent was applied according to the manufacturer’s instructions. The concentration of total RNA was analyzed using Quant-iT RiboGreen (Invitrogen, #R11490, Carlsbad, CA, USA). To assess RNA integrity, samples were analyzed on the TapeStation RNA screentape (Agilent, #5067-5576, Agilent Technologies, Santa Clara, CA, USA). RIN scores greater than 7.0 were used for library preparation. For library construction, 1 μg of total RNA from each sample was prepared with the Illumina TruSeq Stranded mRNA Sample Prep Kit (Illumina Inc., San Diego, CA, USA). The quality of 101 bp paired-end sequencing reads produced from Illumina instruments were assessed using FastQC (version 0.11.7). RNA fragments were reversely transcribed with SuperScript II reverse transcriptase (Invitrogen, #18064014, Carlsbad, CA, USA) random primers to first-strand cDNA. The cDNA underwent end repair, adding the ligation of adapters and single ‘A’ bases. The libraries were quantified using KAPA Library Quantification kits (Illumina sequencing platforms), based on the KAPA BIOSYSTEMS qPCR Quantification Protocol Guide (#KK4854). Library quality was assessed with the TapeStation D1000 ScreenTape (Agilent Technologies, #5067-5582, Santa Clara, CA, USA). Indexed libraries were then sequenced on an Illumina NovaSeq X (Illumina, Inc., San Diego, CA, USA) platform with paired-end (2 × 100 bp) reads.

Before starting analysis, Trimmomatic (version 0.38) [18] was used to eliminate adapter sequences and bases with quality of base lower than 3 from the end reads. To eliminate adapter sequences and low quality, we pre-treated the raw reads from the sequencer upon the *Oncorhynchus mykiss* (USDA_OmykA_1.1) using HISAT v2.1.0(1). HISAT uses two indexes for alignment. The two indexes are constructed utilizing the same Burrows–Wheeler transform, a graph FM index (GFM), as Bowtie2. The reference genome sequence of *Oncorhynchus mykiss* (USDA_OmykA_1.1) and annotation data were obtained in NCBI. Abundance estimation and transcript assembly were performed with StringTie(2, 3).

The expression profile, quantified by the HISAT-StringTie pipeline, was used. The filtered data were log_2_-transformed and normalized with the TMM method.

### 2.4. RT-PCR Analysis Process

#### 2.4.1. Syber Green PCR Analysis

RNA was extracted using QIAzol solution, and the quality of the extracted RNA was assessed by measuring the A260/280 ratio (2.0–2.12), A260/230 ratio (1.69–2.06), RIN value (9.8–10), and rRNA ratio (1.7–1.9). cDNA was synthesized using the Toyobo ReverTra Ace qPCR RT Kit (Toyobo, TOFSQ-101) according to the manufacturer’s instructions. Primer specificity was confirmed by a single band expression through primer testing. RNA expression levels were analyzed using a QuantStudio 12K Flex Sequence Detection System (ThermoFisher, Waltham, MA, USA) on 384-well microtiter plates with a final reaction volume of 10 μL. Amplification was initiated with a 10-min denaturation step at 95 °C, followed by 40 cycles consisting of 10 s at 95 °C, 15 s at 56 °C, 60 °C, or 70 °C depending on the primer set’s melting temperature (Tm), and 30 s at 72 °C. All reactions were performed three times, and with QuantStudio 12K Flex software (ThermoFisher, Waltham, MA, USA). The target genes expression levels were calculated using the comparative Ct (ΔΔCt) method [19]. The primer sequence and relevant information are noted in Table 1.

#### 2.4.2. Taqman PCR Analysis

In the primer test of SYBR green analysis, the metallothionein primer did not pass the quality criteria. However, metallothionein plays an important role in metal stress elimination, and so this study performed additional RNA analysis using Taqman only for metallothionein. RNA extraction and cDNA synthesis were performed using the same method in SYBR green PCR analysis. PCR was performed in a QuantStudio 12K Flex Sequence Detection System (ThermoFisher, Waltham, MA, USA) using 384-well microtiter plates (final volume of 10 μL). We used 5 μL Universal Master Mix (Applied Biosystems, Foster City, CA, USA) and 0.5 uL of 20X Custom TaqMan™ Gene Expression Assay, FAM (Assay ID: APWC7R3; Applied Biosystems, Waltham, MA, USA) as reaction conditions. The assay was performed using an endogenous control, Taqman mRNA TROUT_ACTIN Assay (Assay ID: APU7D66; Applied Biosystems, Waltham, MA, USA). PCR reaction was conducted starting with a 10 min template denaturation step at 95 °C, followed by 40 cycles at 95 °C for 15 s and 60 °C for 1 min three times. Then data were assessed using Quantstudio 12K Flex software (ThermoFisher, Waltham, MA, USA). The Taqman primer sequence and relevant information is noted in Table 2.

#### 2.4.3. Statistical Analysis for RNA Sequencing and RT-PCR Analysis

For RNA sequencing results, significance of statistically differential expression was analyzed with the edgeR exactTest. The false discovery rate (FDR) was controlled through adjusting *p*-values based on the Benjamini–Hochberg procedure. Differentially expressed (DE) genes were identified based on criteria of |fold change| ≥ 2 and raw *p* < 0.05. Gene enrichment, functional annotation, and pathway analyses for the significant gene list were conducted using gProfiler (https://biit.cs.ut.ee/gprofiler/orth (accessed on 1 December 2023)).

For RT-PCR analysis results, the gene expression differences between control and copper-treated cell lines were analyzed statistically using MS Excel (Microsoft Corporation, Washington, DC, USA). Measured results are presented as means ± standard error. Significant differences between the test groups were presented using Student’s *t*-test. *p*-values less than 0.05 was considered to be statistically significant.

## 3. Results

### 3.1. Cytotoxicity Analysis

**Figure 1:**
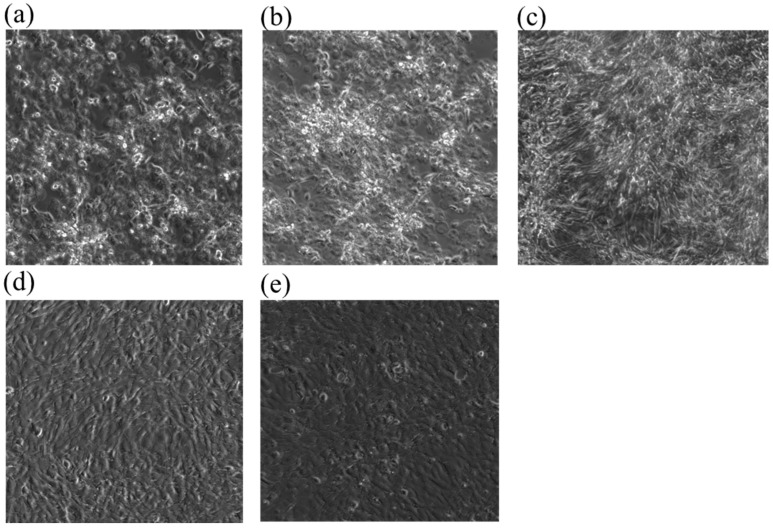
Monolayer morphology variation of RTgill-W1 cell lines treated with copper of (**a**) 1 mg/L-a, (**b**) 1 mg/L-b, (**c**) 400 μg/L, (**d**) 64 μg/L and (**e**) control.

In this study, the RTgill-W1 cell line was exposed to copper at 1 mg/L, 400 μg/L, 160 μg/L, 64 μg/L, 25.6 μg/L and 10.24 μg/L in nominal concentration. Among these concentrations, 1 mg/L, 160 μg/L, 25.6 μg/L, 10.2 μg/L were analyzed with ICP-MS. The measured concentrations were 773.8 μg/L, 131.1 μg/L, 21.4 μg/L, 6.04 μg/L of copper at time 0 and at time 24 h, 683 μg/L, 113.7 μg/L, 17.9 μg/L, 9.57 μg/L, respectively. The EC50 of cell lines stained with AlamarBlue TM (Ala), CFDA-AM (Cfd), Neutral Red (Red) were 549.12 mg/L (Ala), 187.44 mg/L (Cfd), 451.02 mg/L (Red), respectively in nominal concentration. The EC50 of cell lines stained with AlamarBlue TM (Ala), CFDA-AM (Cfd), and Neutral Red(Red) were 507.67 mg/L (Ala), 110.73 mg/L (Cfd), and 451.61 mg/L (Red), respectively in measured concentrations (Table 3). In the analysis of cell morphology, copper stress disrupts the monolayer morphology of cell lines relative to control in a concentration-dependent manner (Figure 1).

### 3.2. RNA Sequencing

#### 3.2.1. GO Profile Analysis

**Figure 2:**
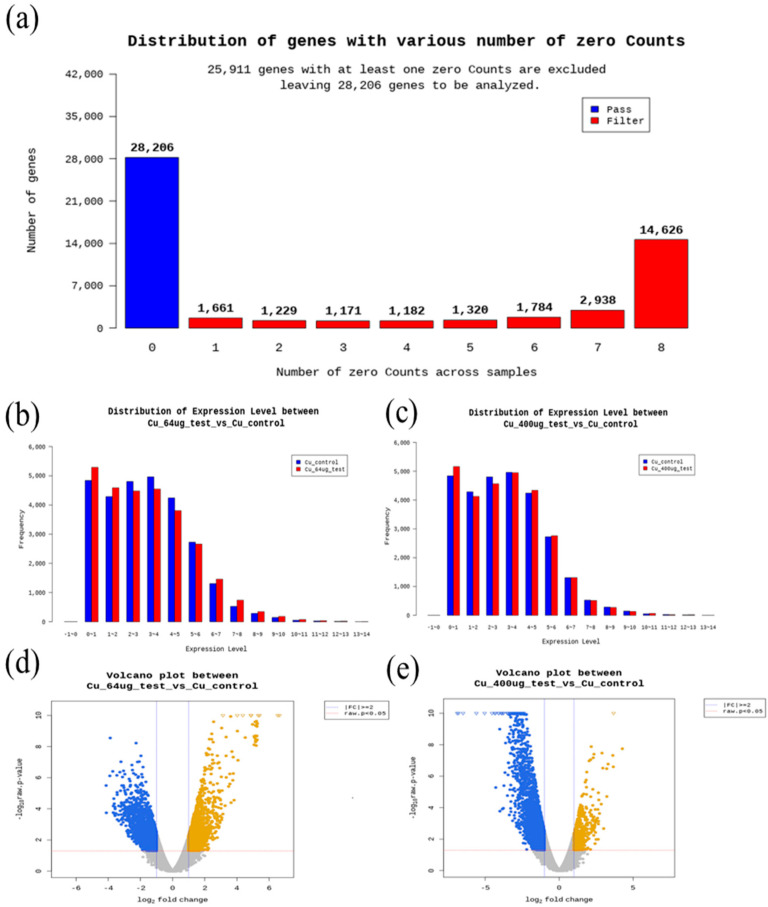
Data quality analysis results: (**a**) distribution of genes with various numbers of zero counts, (**b**) distribution of expression level between 64 μg/L and control, (**c**) distribution of expression level between 400 μg/L and control, (**d**) volcano plot between 64 μg/L and control, (**e**) volcano plot between 400 μg/L and control.

**Figure 3:**
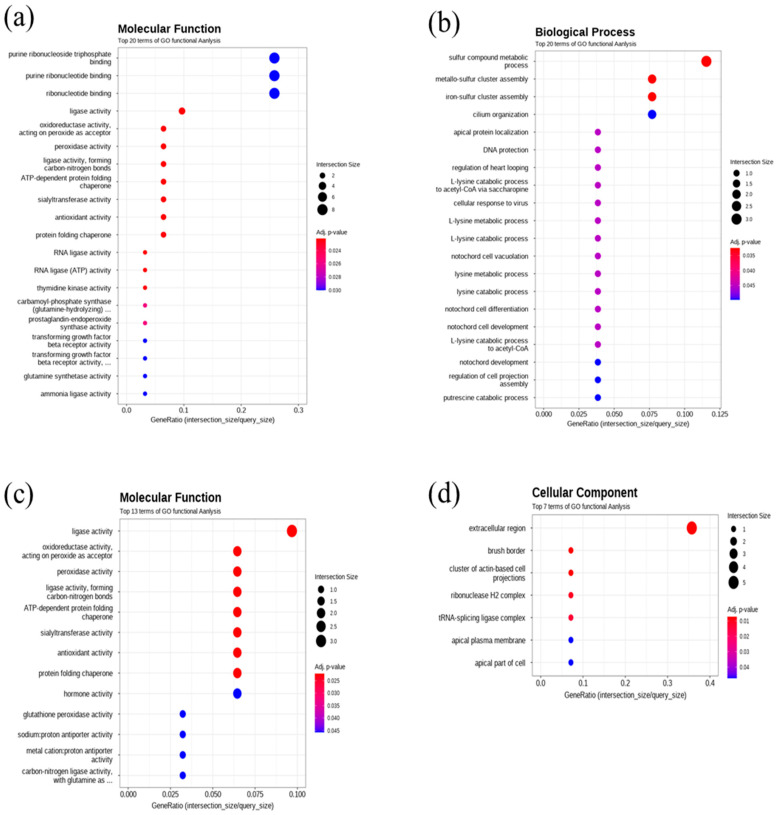
Gene ontology enrichment analysis results: (**a**) GO enrichment in molecular function at 400 μg/L (**b**) in biological process at 64 μg/L, (**c**) in molecular function at 64 μg/L, (**d**) in cellular components at 400 μg/L.

In this work, the quality check of RNA-seq. data is described in Figure 2. Gene ontology and GO enrichment profile analysis results are described in Figure 3. Regarding molecular function terms, after 400 μg/L Cu exposure, we found GO enrichment for purine ribonucleoside triphosphate binding, purine ribonucleotide binding, ribonucleotide binding, ligase activity, oxidoreductase activity, peroxidase activity, ligase activity (carbon-nitrogen bonds), ATP-dependent protein folding chaperone, sialyltransferase activity, antioxidant activity, protein folding chaperone, RNA ligase activity, thymidine kinase activity, carbamoyl phosphate synthase, prostaglandin endoperoxide synthase activity, transforming growth factor beta receptor activity, transforming growth factor beta receptor activity, glutamine synthase activity ammonia ligase activity. Regarding cellular components, after 400 μg/L copper treatmemt, GO enrichment for extracellular region, tRNA-splicing ligase complex, brush border, cluster of actin-based cell projections, apical part of cell, apical plasma membrane, and ribonuclease H2 complex was found. In terms of biological processes, after 400 μg/L Cu treatment, iron-sulfur cluster assembly, sulfur compound metabolic process, metallo-sulfur cluster assembly, L-lysine catabolic process, L-lysine metabolic process, cellular response to virus, L-lysine catabolic process to acetyl-CoA via saccharopine, regulation of heart looping, DNA protection, and apical protein localization were enriched in DEGs compared to the control in the gprofiler analysis results (Figure 3).

#### 3.2.2. Major Differentially Expressed Genes in RNA Seq

In response to 64 and 400 μg/L copper, the genes relevant to growth, proliferation and differentiation of cell/embryo/organ were significantly increased compared to the control. In detail, *growth/differentiation factor 10*, *IGF-like family receptor*, *G1/S-specific cyclin-D1*, and others were significantly upregulated, whereas *proto-oncogene c-fos*, *early growth response* (*ERG*) *protein*, *G0/G1 switch protein*, *DEP domain-containing mTOR interacting protein* (*DEPTOR*), *angiopoietin related protein*, and *aurora kinase* were down-regulated.

Immune function, apoptosis, oxidative stress, and inflammation-related genes were also significantly regulated. TNF receptor-associated factor2 (TRAFs), leukocyte surface antigen CD53, VHSV-induced protein 5, eotaxin, aifm4, bcl2a, carbonic anhydrase 4 (CA4), SOD1, GSTt1a, Sirtuin 1, and Sirtuin 4 were significantly changed. In terms of energy metabolism genes and xenobiotic metabolism, apolipoprotein E, glucose 6-phosphate 1-dehydrogenase, ATP-binding cassette sub family 1, and others showed a significant change.

### 3.3. RT-PCR Analysis

The genes recognized to be significantly changed by copper exposure in the RNA sequencing were analyzed by RT-PCR analysis, to suggest effective biomarkers for copper exposure. The analyzed genes were chosen from among the genes showing statistically significant changes relative to control. Among them, the genes satisfying the primer test were chosen as the target genes.

**Figure 4:**
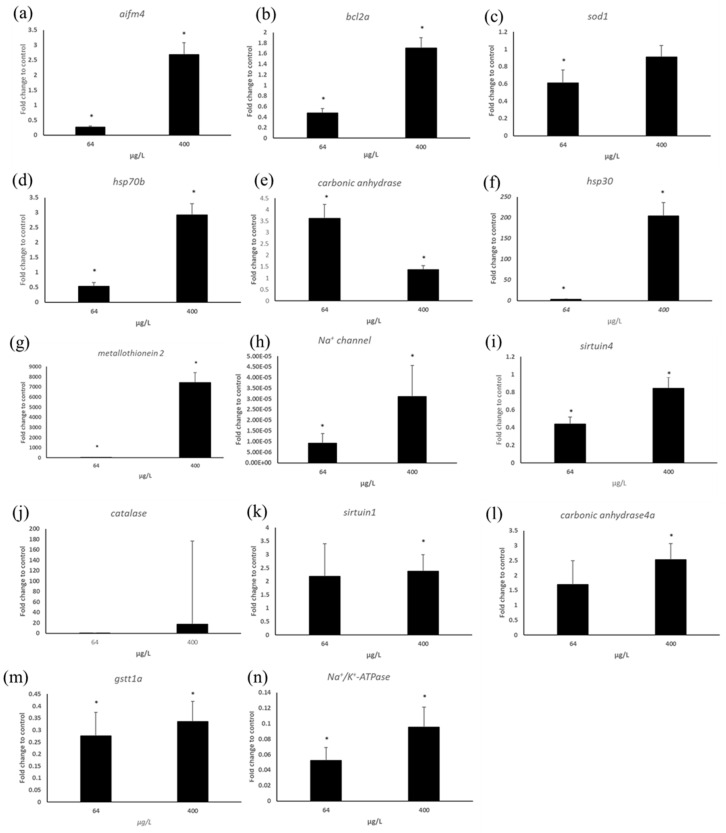
RT-PCR analysis results for genes such as (**a**) *aifm4* (**b**) *bcl2a*, (**c**) *sod1*, (**d**) *hsp70b*, (**e**) *carbonic anhydrase*, (**f**) *hsp30*, (**g**) *metallothionein2*, (**h**) *Na^+^ channel*, (**i**) *sirtuin4*, (**j**) *catalase*, (**k**) *sirtuin1*, (**l**) *carbonic anhydrase4*, (**m**) *gstt1a*, (**n**) *Na^+^/K^+^ ATPase.* Star mark indicates significance compared to control (* *p* < 0.05).

In response to 400 μg/L copper, the metal stress-related gene *metallothionein 2* showed significant upregulation, while 64 μg/L copper exposure caused down-regulation (Figure 4). Chaperone protein gene *hsp30* expression was increased at both concentrations relative to the control, while *hsp70b* gene expression was increased only at 400 μg/L. The ion channel protein gene *Na^+^/K^+^-ATPase* was significantly down-regulated at both concentrations. Similarly, the *Na^+^ channel* gene showed significant down-regulation relative to the control. *Carbonic anhydrase* showed 3.5-fold induction relative to the control at 64 μg/L, and at 400 μg/L its expression level was 1.5-fold increased relative to the control group. *Carbonic anhydrase 4* (CA4) gene expression level was increased at both concentrations, whereas, similar with *carbonic anhydrase*, the expression level of 64 μg/L was higher than 400 μg/L (Figure 4).

The expression level of *sirtuin* genes related to energy metabolism, apoptosis, tumor suppress, insulin secretion, and others was significantly changed. *Sirtuin 4* gene was down-regulated at both concentrations, whereas at 400 μg/L there was no significant change relative to the control. *Sirtuin* 1 showed a 2.5-fold increase at both concentrations relative to the control. Oxidative stress-related genes were also changed in their expression levels. The expression level of *SOD1*(Cu-Zn) was reduced at both concentrations. Similarly, *GSTt1a* was down-regulated at both concentrations. In case of *catalase*, the average-fold induction level related to the control was increased, but due to higher standard error, there was no significant change at both copper concentrations, while in RNA seq., the level was decreased by 3-fold at 64 μg/L.

Apoptosis-related genes also showed significant changes. *aifm4* (apoptosis-inducing factor 4) showed a 2-fold induction at 400 μg/L while the level was decreased by 0.2-fold at 64 μg/L. *BCL2a* gene expression was significantly increased at 400 μg/L, while at 64 μg/L the expression level was reduced at 64 μg/L (Figure 4).

## 4. Discussion

### 4.1. Cellular Toxicity

The 24h-EC50 values that could be used as predicted values replacing the 96h-LC50 values of the fish test aligned well with previously reported LC50 toxicity ranges for fish. In detail, an LC50 of 0.094 mg/L was observed in rainbow trout exposed to copper [20], and other studies showed an LC50 of 0.539 mg/L at 1.5 g and 0.44 mg/L at 3.5 g for rainbow trout [21]. In this work, for Cfd analysis, EC50 had higher toxicity than Alr and Red, meaning that copper stress affected cell membrane integrity rather than lysosomal membrane (Red) or metabolic activity (Alr). However, RTgill-W1 cells exposed to Cu^2+^ showed an EC50 (Cfd dye, 2.5 h, pH7) value of 0.63 μM (39.69 mg/L) [22]. This discrepancy could be due to the 1 h exposure time and difference in experimental medium, as Earle’s medium was used instead of L15 medium. In terms of cell line morphology, at 1000 and 400 μg/L of copper, disruption of confluent cell line morphology was found under the microscope. However, cell lines treated with 160 μg/L copper or below, along with the control cell line, did not show any morphological change, meaning at about EC50 value, morphological change can be observed (Figure 1).

These findings suggest that toxicity predictions based on RTgill-W1 cell line tests are consistent with in vivo fish toxicity results, indicating that the cell line can be a reliable indicator of copper toxicity. Additionally, measured copper concentrations in the experiments were lower than nominal concentrations. This may be due to interactions with media components from FBS or L15 exposure media, although the exact reason is not known. This discrepancy underscores the need for further studies to clarify the effects of media chemistry on copper bioavailability and concentration accuracy.

### 4.2. Gene Expression Profiles in RT-PCR and RNA Sequencing

For the gene expression analysis results, this work discuss the findings in the following order: osmoregulatory disturbance and Na^+^/K^+^-ATPase activity, oxidative stress and apoptosis, energy metabolism, stress regulation, immune function change, and the MAPK pathway.

Copper has been known to cause various effects, including osmoregulatory disturbances, changes in energy balance such as glucose levels, regulation of Na^+^/K^+^-ATPase activity, oxidative stress, apoptosis in tissues like gills, liver, kidneys, gonads, and brain, as well as behavioral changes [16]. One notable effect is osmoregulatory disturbance, which may result from copper’s influence on ion regulation and enzyme activity. Exposure of amphipods (*Gammarus pulex*) to copper caused a reduction in sodium concentration and sodium influx in hemolymph [23]. Waterborne copper at 40 and 400 μg/L caused dysregulation of gill Na^+^/K^+^-ATPase activity, plasma Na^+^, Cl^−^, osmolality, protein, glucose, and cortisol levels [24]. Copper inhibited Na^+^/K^+^-ATPase activity in the gills of *Callinectes sapidus*, suggesting disruption of salt and water balance regulation by this enzyme [25]. A decrease in plasma osmolality was observed in the gills of *Rhamdia quelen* exposed to 11 μg/L of copper, with chloride content decreasing at 2 μg/L [26]. Conversely, in *Cyprinus carpio* exposed to cortisol, there were increases in Na^+^/K^+^-ATPase activity and plasma osmolarity, but these levels decreased following copper treatment. Previous research showed that copper inhibits Na^+^ ion efflux in fish gills, leading to changes in Na^+^/K^+^-ATPase activity [27]. RNA sequencing results in this work revealed that the gene expression of *Na^+^/K^+^-ATPase, aquaporin 10b*, *chloride channel 3*, and *sodium channel subunit beta-1* was significantly reduced, while chloride channel 7 and sodium channel type 4 were increased. In the GO profile, the extracellular region and apical plasma membrane were found to be enriched by copper exposure (Figure 3). Additionally, RT-PCR analysis indicated that *Na^+^/K^+^-ATPase* and *sodium channel* expression were changed. These findings suggest that copper disturbs metal ion regulation in organisms. Considering that cell membrane integrity can be affected by osmosis and water balance around the cell membrane, the significant osmoregulatory changes support cell viability test results showing higher cell membrane integrity damage than other effects (Table 1). Furthermore, RTgill-W1 cell lines can serve as an effective system for predicting copper-induced osmoregulatory changes.

Copper toxicity is considered to be induced by oxidative stress, followed by apoptosis and other physiological malfunctions. In fact, RTgill-W1 cells showed induction of ROS and DNA strand breaks for 6.8 × 10^−10^, 2.16 × 10^−8^, 5.39 × 10^−8^ M of copper exposure within 1 h, suggesting that copper causes oxidative stress in the RTgill-W1 cell line [22]. Previously, the adverse effects of copper on organisms were found to result from the indirect production of reactive oxygen species (ROS) [28]. In response to copper treatments, the liver of javelin goby (*Synechogobius hasta*) showed significant changes in the expression of apoptosis-related genes associated with oxidative stress. Genes such as *casp3*, *dff45*, *dff40*, *ikk*, *ikba*, *nfkb*, and *pI3k* were decreased, while calpain was increased [12]. When exposed to copper, the liver of *Oncorhynchus mykiss* exhibited alterations in amino acid metabolism, including reductions in serine and arginine levels, an increase in proline content, and inhibition of apoptosis. The decrease in serine and arginine suggests a reduction in antioxidant capacity, whereas the increase in proline and induction of apoptosis may contribute to copper ion resistance in this species [15]. Copper exposure caused significant increases in gene expression of *sod*, *cp*, and *cat*, which later decreased to basal levels. Furthermore, copper exposure induced histophysiological changes in the liver and promoted cell apoptosis [29]. In this study, RNA sequencing results showed that markers of oxidative stress were significantly changed, with fluctuations in apoptosis-related gene expression. Specifically, the *sod1* gene was significantly increased in the transcriptome analysis while the gene expression of *gst theta* was significantly downregulated in both PCR and RNA-seq. In the literature, oxidative stress can lead to both downregulation and upregulation of antioxidant genes simultaneously, which may be related to an overwhelmed antioxidant capacity or oxidative stress-induced malfunction of antioxidant enzymes [3,4]. Previous studies have indicated that the expression of *sirtuin* 1 and 4 can be altered by oxidative stress, affecting apoptosis [30]. In this study’s transcriptome analysis, *Sirtuin 1* was significantly decreased, while *Sirtuin 4* was increased. Interestingly, in RT-PCR, *Sirtuin 1* and *Sirtuin 4* expression increased significantly. The apoptosis-related gene *aifm4* showed a significant increase compared to the control. Supporting this, the *bcl2a* gene, related to apoptosis, showed significant changes; specifically, in RNA-seq., *bcl2a* was significantly upregulated at the highest concentration. Overall, these results support the idea that copper causes oxidative stress, which subsequently triggers apoptosis in response to copper exposure.

Copper exposure can cause changes in energy metabolism related to glucose, fatty acid, and amino acid metabolism. As supporting data, previous studies on *Prochilodus lineatus* have revealed that liver enzymes such as *phosphofructokinase*, *pyruvate kinase*, *hexokinase*, *lactate dehydrogenase*, and *glucose-6-phosphate dehydrogenase* showed significant changes in response to copper, indicating substantial alterations in carbohydrate metabolism [11]. Waterborne copper at 40 and 400 μg/L caused dysregulation of gill *Na^+^/K^+^-ATPase* activity participating in energy generation, protein, glucose, and cortisol levels [24]. In other in vivo studies, copper exposure affected energy balance as well. *Golden trout* exposed to 60 and 120 μg/L copper for 96 h showed significant changes in *glucose-6-phosphate dehydrogenase*, *phosphoenolpyruvate carboxykinase*, and NADPH/NADP^+^ ratios. At 120 μg/L, NAD^+^, ATP content, glycolytic pathway activity, and mitochondrial respiratory chain complex I activity were downregulated [13]. Copper binds to fatty acylated components in the TCA cycle, leading to aggregation and imbalance of these proteins, inhibiting the cycle, inducing stress, and ultimately causing cell death [14]. Copper exposure also caused a reduction in hepatic ATP activity [15]. Similarly, Chen et al. (2022) [13] observed an increased NADH/NAD^+^ ratio with decreased ATP levels and elevated MDA levels at 120 μg/L copper. Lipid metabolism processes, including fatty acid degradation, steroid biosynthesis, fatty acid elongation, and glycerophospholipid metabolism, were significantly affected, especially fatty acid β-oxidation-related genes such as *long-chain acyl-CoA synthetase* (*acsl*), *carnitine O-palmitoyltransferase 1* (*cpt1*), *enoyl-CoA hydratase* (*ech*), and *acetyl-CoA acyltransferase* (*acaa*) [12]. In fact, transcriptome analysis of this work showed that genes such as *glucose-6-phosphate dehydrogenase*, *hexokinase*, *ATP-dependent 6-phosphofructokinase*, *fatty acid desaturase*, *fatty acid elongation protein 4*, and *Na^+^/K^+^-ATPase* were significantly downregulated. Similarly, RT-PCR analysis revealed that *Na^+^/K^+^-ATPase*, which plays a role in ion transfer across membranes and ATP synthesis, was significantly downregulated. Furthermore, *sirtuin 1* and *4*, involved in insulin secretion, fatty acid oxidation, amino acid metabolism, and other processes, exhibited significant regulation in response to copper stress in RNA-seq. *Sirtuin 1* and *sirtuin 4* were significantly up- or downregulated, as confirmed by PCR analysis.

Additionally, copper stress caused significant fluctuations in stress-related genes involved in cellular compensation. Previously chaperone proteins such as HSP70/30/70b were shown to inhibit protein unfolding and aggregation, assist in protein folding, and maintain homeostasis under oxidative stress and heat shock [31,32,33]. Previous studies have reported that copper exposure increased *hsp70* expression in rainbow trout hepatocytes, which was associated with lactate dehydrogenase leakage and increased apoptosis, effects that were mitigated by ROS scavengers like vitamin C, indicating the protective role of HSP70 against oxidative stress [32]. The regulation of HSP proteins under chemical stress likely reflects responses to protect or refold damaged proteins. In yeast, *haa1* regulates genes such as *yro2* (hsp30 orthologue in yeast), a copper-regulated transcription factor, suggesting copper exposure can induce *hsp30* gene regulation [34]. Also, copper can cause upregulation of metallothionein expression in rainbow trout gills to detoxify metal toxicity and maintain cellular metal homeostasis [35]. In this study, RNA-seq analysis showed enrichment of DNA ligase, RNA ligase activity, and protein folding chaperone, suggesting involvement in repair, splicing, and editing of RNA, DNA, and proteins, likely related to copper-induced damage to RNA and others, and diverse gene expression responses (Figure 3). RT-PCR analysis showed that *hsp70b* was significantly downregulated by copper at both concentrations, while RNA-seq at 64 μg/L indicated upregulation. *Hsp30*, on the other hand, was upregulated at both concentrations in RT-PCR analysis. This work showed that copper caused a significant increase of metallothionein2 gene expression at 400 μg/L.

Metalloprotein expression was also significantly affected by copper exposure. Previously, branchial carbonic anhydrase activity was found to be downregulated at 2 μg/L of copper [26]. This study consistently demonstrated that carbonic anhydrase activity changed significantly. In RT-PCR analysis, carbonic anhydrase—which is involved in acid-base regulation, respiration, and bone metabolism—showed a significant increase at both concentrations. In RNA-seq analysis, carbonic anhydrase 4 activity showed a significant 2-fold decrease at 400 μg/L, while the level was upregulated at 64 μg/L. Transcriptome analysis also showed that sulfur compound metabolic process and metallo-sulfur cluster assembly were enriched at 64 μg/L copper exposure (Figure 3).

Copper also plays a role in change in the immune system [16]. A previous study mentioned that oxidative stress can cause the release of inflammatory mediators and immune function damage [36]. In response to copper exposure, the liver of *Synechogobius hasta* (javelin goby) showed an immune response. Genes involved in immune pathways, such as chemokine signaling, B cell receptor signaling, and complement and coagulation cascades, were affected: *Crc*, *pi3k*, and *ikk* were decreased; *TfpI*, *a2m*, *kng*, *hf1*, *bf*, *df*, *mcp*, and *c1q* were increased, while *c1r*, *c7*, and *c9* were decreased [12]. Copper upregulated immune-related gene expression in the liver, including *hsp90*. Additionally, *il-1B* showed a significant increase on the second day, then returned to baseline during recovery [15]. Copper exposure caused histophysiological changes in the liver and induced cell apoptosis. Furthermore, copper initially increased LZM activity, which decreased over time [29]. In this work, RNA-seq analysis showed the gene expression level of *interleukin 11*, *T-cell activation Rho GTPase-activating protein* and others involved in the immune system were significantly changed.

Regarding signal transduction, the MAPK signaling pathway—including *fgf*, *fgfr*, *cpla2*, and *c-fos*—was upregulated, while *cacn*, *grb2*, and *ikk* were downregulated. The calcium signaling and NFκB pathways were also enriched [12]. Similarly, RNA-seq analysis showed that *MAPK interacting serine/threonine kinase 1/2* decreased.

In summary, based on RNA-seq and RT-PCR results, copper exposure induces oxidative stress and alters ion content, and this triggers a cascade of molecular events. These include antioxidant activation, regulation of osmolarity-related genes, apoptosis, MAPK pathway activation, stress-induced protein expression, immune response modulation, and energy metabolism changes. These molecular responses contribute to copper-induced adverse effects at higher biological organization levels, such as organs and individuals. This study identified several promising biomarkers that significantly change in gene expression following copper treatment, including *sirtuin 1*, *sirtuin 4*, *Na^+^/K^+^-ATPase*, *carbonic anhydrase*, *Na^+^ channel*, *hsp30*, *hsp70b*, *aifm4*, and others, based on existing literature and this study’s results of RNA sequencing and PCR analysis. Finally, the study demonstrates that RTgill-W1 cell lines are an effective alternative system for copper toxicity testing, potentially replacing *Oncorhynchus mykiss* in future assessments.

## Figures and Tables

**Table 1:** Information of primer used in SYBR green PCR analysis.

Gene	Product Size (bp)	Tm	Forward	Reverse
*hsp30*	88	65	ACGGGAAGCTCCACATTCAG	AGGCTACAGTTGATGGGCAC
*hsp70b*	90	65	GAGAAGAGATACGTTCAACATTCA	GGTCAATGCCGATAGACGG
*catalase*	148	70	AACAACCACCACTGTTTCCT	ACTTCTCTTCAGACAGGCGA
*gstt1a*	127	70	GTAGAGATCATGCAGCCGGT	TGGTCTTGTGAGCTTCGTCA
*sod1*	125	65	GGACTTTGGGAACTTTGTGGC	GGTCGTCTGCCTTCTCATGG
*sirt4*	96	65	CTCGTGCCATTGTAAGATGCG	ATGCCCTCGGGAACTGATGA
*sirtuin1*	85	70	CTTTGCCTTCCCAGATGGTG	TATCTGTGGTTGAGGGGTCC
*aifm4*	104	65	AAGGCACAGGCACAATCTGA	GTTAAAGGAGGTACCGTCGGA
*bcl2a*	148	65	TTCCGTCTTTCTGGAACTGTGT	GTCAACATGAGCGTCCGAGA
*Na^+^ channel*	83	70	AGACGGCTGACGATGTTATG	CGTGTTGATCTCACAGCACT
*carbonic anydrase*	88	65	TGCTGAGGGTTTAGTGACGG	GGATCAAAGTGAACCCTGTTGG
*ca4a*	101	70	TGGACTGTGTTCGAGAAGCC	CGGTATGTGCCCACCATAGG
*Na^+^/K^+^* *-* *ATPase*	139	70	CACAGCAAAGAGGGACACACC	CAGCAACAGCTTAACCGCAAC
*B-actin*	82	65	CACAACTGGAACGGTGAAGC	GAATCTCAGGGGTCCTTTTACA

**Table 2:** Information of primer used in Taqman PCR analysis.

Gene	Reporter 1 Dye	Reporter 1 Quencher	Forward Sequence	Reverse Sequence	Reporter 1 Sequence	Context Sequence
*B-actin*	FAM	NFQ	GCCCAGAGGCCCTCTTC	CTCGTGGATACCGCAAGACT	CAGCCCTCCTTCCTCG	GCCCAGAGGCCCTCTTCCAGCCCTC
*Metallothionein2*	FAM	NFQ	CGCATGCACCAGTTGTAAGAAAG	GCCTGAGGCACACTTGCT	CAGTCGCAGCAACTTG	GCAAGTTGCTGCGACTGCTGTCCCT

**Table 3:** EC50 value of copper exposure in toxicity test using the RTgill-W1 cell line in nominal concentration and measured concentration.

	EC50 (μg/L)	95% Confidence Interval (μg/L)
Nominal con.—Cu-Ala	549	482–615
Nominal con.—Cu-Cfd	187	166–208
Nominal con.—Cu-Red	451	409–492
Measured con.—Cu-Ala	507	453–561
Measured con.—Cu-Cfd	110	97–124
Measured con.—Cu-Red	451	407–495

## Data Availability

The data used in this work is included in the article.
